# COVID-19 Coronavirus Vaccine Design Using Reverse Vaccinology and Machine Learning

**DOI:** 10.3389/fimmu.2020.01581

**Published:** 2020-07-03

**Authors:** Edison Ong, Mei U Wong, Anthony Huffman, Yongqun He

**Affiliations:** ^1^Department of Computational Medicine and Bioinformatics, University of Michigan, Ann Arbor, MI, United States; ^2^Unit for Laboratory Animal Medicine, Department of Microbiology and Immunology, University of Michigan, Ann Arbor, MI, United States

**Keywords:** COVID-19, S protein, non-structural protein 3, vaccine, reverse vaccinology, machine learning, vaxign, vaxign-ML

## Abstract

To ultimately combat the emerging COVID-19 pandemic, it is desired to develop an effective and safe vaccine against this highly contagious disease caused by the SARS-CoV-2 coronavirus. Our literature and clinical trial survey showed that the whole virus, as well as the spike (S) protein, nucleocapsid (N) protein, and membrane (M) protein, have been tested for vaccine development against SARS and MERS. However, these vaccine candidates might lack the induction of complete protection and have safety concerns. We then applied the Vaxign and the newly developed machine learning-based Vaxign-ML reverse vaccinology tools to predict COVID-19 vaccine candidates. Our Vaxign analysis found that the SARS-CoV-2 N protein sequence is conserved with SARS-CoV and MERS-CoV but not from the other four human coronaviruses causing mild symptoms. By investigating the entire proteome of SARS-CoV-2, six proteins, including the S protein and five non-structural proteins (nsp3, 3CL-pro, and nsp8-10), were predicted to be adhesins, which are crucial to the viral adhering and host invasion. The S, nsp3, and nsp8 proteins were also predicted by Vaxign-ML to induce high protective antigenicity. Besides the commonly used S protein, the nsp3 protein has not been tested in any coronavirus vaccine studies and was selected for further investigation. The nsp3 was found to be more conserved among SARS-CoV-2, SARS-CoV, and MERS-CoV than among 15 coronaviruses infecting human and other animals. The protein was also predicted to contain promiscuous MHC-I and MHC-II T-cell epitopes, and the predicted linear B-cell epitopes were found to be localized on the surface of the protein. Our predicted vaccine targets have the potential for effective and safe COVID-19 vaccine development. We also propose that an “Sp/Nsp cocktail vaccine” containing a structural protein(s) (Sp) and a non-structural protein(s) (Nsp) would stimulate effective complementary immune responses.

## Introduction

The emerging Coronavirus Disease 2019 (COVID-19) pandemic poses a massive crisis to global public health. As of March 11, 2020, there were 118,326 confirmed cases and 4,292 deaths, according to the World Health Organization (WHO), and WHO declared the COVID-19 as a pandemic on the same day. On May 12, WHO reported 4,088,848 confirmed COVID-19 cases and 283,153 deaths globally, showing a dramatic increase in terms of case and death numbers. The causative agent of the COVID-19 disease is the severe acute respiratory syndrome coronavirus 2 (SARS-CoV-2). Coronaviruses can cause animal diseases such as avian infectious bronchitis caused by the infectious bronchitis virus (IBV), and pig transmissible gastroenteritis caused by a porcine coronavirus (1). Bats are commonly regarded as the natural reservoir of coronaviruses, which can be transmitted to humans and other animals after genetic mutations. There are seven known human coronaviruses, including the novel SARS-CoV-2. Four of them (HCoV-HKU1, HCoV-OC43, HCoV-229E, and HCoV-NL63) have been circulating in the human population worldwide and cause mild symptoms (2). Coronavirus became prominent after Severe Acute Respiratory Syndrome (SARS) and Middle East Respiratory Syndrome (MERS) outbreaks. In 2003, the SARS disease caused by the SARS-associated coronavirus (SARS-CoV) infected over 8,000 people worldwide and was contained in the summer of 2003 (3). SARS-CoV-2 and SARS-CoV share high sequence identity (4). The MERS disease infected more than 2,000 people, which is caused by the MERS-associated coronavirus (MERS-CoV) and was first reported in Saudi Arabia and spread to several other countries since 2012 (5).

Great efforts have been made to develop and manufacture COVID-19 vaccines, and these efforts in pushing the vaccine clinical trials are phenomenal (Table 1). Coronaviruses are positively-stranded RNA viruses with its genome packed inside the nucleocapsid (N) protein and enveloped by the membrane (M) protein, envelope (E) protein, and the spike (S) protein (6). While many coronavirus vaccine studies targeting different structural proteins were conducted, most of these efforts eventually ceased soon after the outbreak of SARS and MERS. With the recent COVID-19 pandemic outbreak, it is urgent to resume the coronavirus vaccine research. As the immediate response to the on-going pandemic, the first testing in humans of the mRNA-based vaccine targeting the S protein of SARS-CoV-2 (ClinicalTrials.gov Identifier: NCT04283461, Table 1) started on March 16, 2020. As the most superficial and protrusive protein of the coronaviruses, S protein plays a crucial role in mediating virus entry. In the SARS and MERS vaccine development, the full-length S protein and its S1 subunit (which contains receptor binding domain) have been frequently used as the vaccine antigens due to their ability to induce neutralizing antibodies that prevent host cell entry and infection.

**Table 1:** Reported clinical trials of preventive SARS-CoV, MERS-CoV, SARS-CoV-2 vaccine studies.

**Virus**	**Location**	**Phase**	**Year**	**Identifier**	**Vaccine type**
SARS-CoV	United States	I	2004	NCT00099463	Recombinant DNA vaccine (S protein)
SARS-CoV	United States	I	2007	NCT00533741	Inactivated whole virus vaccine
SARS-CoV	United States	I	2011	NCT01376765	Recombinant protein vaccine (S protein)
MERS	United Kingdom	I	2018	NCT03399578	Vector vaccine (S protein)
MERS	Germany	I	2018	NCT03615911	Vector vaccine (S protein)
MERS	Saudi Arabia	I	2019	NCT04170829	Vector vaccine (S protein)
MERS	Germany, Netherland	I	2019	NCT04119440	Vector vaccine (S protein)
MERS	Russia	I, II	2019	NCT04128059	Vector vaccine (protein not specified)
MERS	Russia	I, II	2019	NCT04130594	Vector vaccine (protein not specified)
SARS-CoV2	United States	I	2020	NCT04283461	mRNA-based vaccine (S protein)
SARS-CoV2	China	I	2020	NCT04313127	Vector vaccine (S protein)
SARS-CoV2	China	II	2020	NCT04341389	Vector vaccine (S protein)
SARS-CoV2	China	I, II	2020	NCT04352608	Inactivated whole virus vaccine
SARS-CoV2	United Kingdom	I, II	2020	NCT04324606	Vector vaccine (S protein)
SARS-CoV2	United States	I	2020	NCT04336410	DNA vaccine (S protein)

However, the current coronavirus vaccines, including S protein-based vaccines, might have issues in the lack of inducing complete protection and possible safety concerns (7, 8). Most existing SARS/MERS vaccines were reported to induce neutralizing antibodies and partial protection against the viral challenges in animal models (Table 2). A recent study reported that adenovirus vaccine vector encoding full-length MERS-CoV S protein (ChAdOx1 MERS) showed protection upon MERS-CoV challenge in rhesus macaques (9). Nonetheless, it is desired for a COVID-19 vaccine to induce complete protection or sterile immunity. Moreover, it has become increasingly clear that multiple immune responses, including those induced by humoral or cell-mediated immunity, are responsible for correlates of protection than antibody titers alone (10). Both killed SARS-CoV whole virus vaccine and adenovirus-based recombinant vector vaccines expressing S or N proteins induced neutralizing antibody responses but did not provide complete protection in animal model (11). A study has shown increased liver pathology in the vaccinated ferrets immunized with modified vaccinia Ankara-S recombinant vaccine (12). The safety and efficacy of these vaccination strategies have not been fully tested in human clinical trials, but safety could be a major concern. Therefore, novel strategies are needed to enhance the efficacy and safety of COVID-19 vaccine development.

**Table 2:** Experimentally verified vaccines for SARS-CoV, MERS-CoV, and SARS-CoV-2.

**Vaccine name**	**Vaccine type**	**Antigen**	PMID/doi^*^
**SARS VACCINES**			
CTLA4-S DNA vaccine^**^	DNA	S	15993989
*Salmonella*-CTLA4-S DNA vaccine^**^	DNA	S	15993989
*Salmonella*-tPA-S DNA vaccine^**^	DNA	S	15993989
Recombinant spike polypeptide from *E. coli* vaccine^**^	Recombinant	S	15993989
Recombinant spike polypeptide from insect cells vaccine	Recombinant	S	22536382
pCI-N protein DNA vaccine	DNA	N	15582659
CRT/pcDNA3.1/myc-His(-)N DNA vaccine	DNA	N	15078946
M protein DNA vaccine	DNA	M	16423399
pcDNA3.1/myc-His(-)-N protein DNA vaccine	DNA	N	15078946
pcDNA3.1/myc-His(-)-N+M protein DNA vaccine	DNA	N, M	16423399
tPA-S DNA vaccine^**^	DNA	S	15993989
β-propiolactone-inactivated SARS-CoV vaccine	Inactivated virus	Whole virus	16476986
Dual-inactivated virus (DIV) SARS-CoV vaccine	Inactivated virus	Whole virus	22536382
UV-Inactivated SARS virus vaccine + TLR agonist	Inactivated virus	Whole virus	24850731
MA-ExoN vaccine	Live attenuated	MA-ExoN	23142821
rMA15-ΔE vaccine	Live attenuated	MA15	23576515
rSARS-CoV-ΔE vaccine	Live attenuated	SARS-CoV-ΔE	18463152
VLP SARS-CoV vaccine	Viral-like particle	S,N,E,M	22536382
Ad S/N vaccine	Viral vector	S,N	16476986
ADS-MVA vaccine	Viral vector	S	15708987
MVA/S vaccine	Viral vector	S	15096611
SV8000 vaccine	Viral vector	S, N, ORF8	10.1101/2020.02.17.951939
VRP-SARS-N vaccine^***^	Viral vector	N	27287409
**MERS VACCINES**			
England1 S DNA Vaccine	DNA	S	26218507
MERS-CoV pcDNA3.1-S1 DNA vaccine	DNA	S	28314561
Inactivated whole MERS-CoV (IV) vaccine	Inactivated virus	Whole virus	29618723
England1 S DNA +England1 S protein subunit Vaccine	Mixed	S1	26218507
England1 S1 protein subunit Vaccine^**^	Subunit	S1	26218507
MERS-CoV S vaccine	Subunit	S	29618723
rNTD vaccine	Subunit	NTD of S	28536429
rRBD vaccine	Subunit	RBD of S	28536429
MERS-CoV VLP vaccine	Viral-like particle	S, E, M	27050368
Ad41.MERS-S vaccine^**^	Viral vector	S	25762305
Ad5.MERS-S vaccine^**^	Viral vector	S	25192975
Ad5.MERS-S1 vaccine^**^	Viral vector	S1	25192975
ChAdOx1-MERS-S vaccine	Viral vector	S	29263883
MVvac2-CoV-S(H) vaccine	Viral vector	S	26355094
MVvac2-CoV-solS (H) vaccine	Viral vector	solS	26355094
RVΔP-MERS/S1 vaccine^**^	Viral vector	S1	31589656
VRP-MERS-N vaccine^***^	Viral vector	N	27287409
VSVΔG-MERS vaccine^**^	Viral vector	S	29246504
**SARS-CoV-2 VACCINES**			
PiCoVacc vaccine	Inactivated virus	Whole virus	10.1101/2020.04.17.046375
RBD-CuMVTT vaccine^**^	VLP	RBD	10.1101/2020.05.06.079830
LPN-SARS-Cov-2 vaccine^**^	RNA	S	10.1101/2020.04.22.055608

*S, surface glycoprotein; N, nucleocapsid phosphoprotein; M, membrane glycoprotein; Exon, exoribonuclease; NTD, N-terminal domain; RBD, receptor binding domain; ORF8, open reading frame 8; solS, truncated soluble surface glycoprotein; VLP: Virus-like particles*.

*, *Journal articles have their PMID while pre-print papers have their doi*.

**, *Only have an immune response and not a formal challenge study according to the source*.

***, *This vaccine also gives cross-protection to MERS-CoV or SARS-CoV*.

In recent years, the development of vaccine design has been revolutionized by the reverse vaccinology (RV), which aims to first identify promising vaccine candidate through bioinformatics analysis of the pathogen genome. RV has been successfully applied to vaccine discovery for pathogens such as Group B meningococcus and led to the license Bexsero vaccine (13). Among current RV prediction tools (14, 15), Vaxign is the first web-based RV program (16) and has been used to predict vaccine candidates against different bacterial and viral pathogens (17–19). Recently we have also developed a machine learning approach called Vaxign-ML to enhance prediction accuracy (20).

In this study, we first surveyed the existing coronavirus vaccine development status, and then applied the Vaxign and Vaxign-ML RV approaches to predict COVID-19 protein candidates for vaccine development. We identified six possible adhesins, including the structural S protein and five other non-structural proteins, and three of them (S, nsp3, and nsp8 proteins) were predicted to induce high protective immunity. The S protein was predicted to have the highest protective antigenicity score, and it has been extensively studied as the target of coronavirus vaccines by other researchers. The sequence conservation and immunogenicity of the multi-domain nsp3 protein, which was predicted to have the second-highest protective antigenicity score yet, was further analyzed in this study. Based on the predicted structural S protein and non-structural proteins (including nsp3) using reverse vaccinology and machine learning, we proposed and discussed a cocktail vaccine strategy for rational COVID-19 vaccine development.

## Results

### Published Research and Clinical Trial Coronavirus Vaccine Studies

To better understand the current status of coronavirus vaccine development, we systematically surveyed the development of vaccines for coronavirus from the ClinicalTrials.gov database and PubMed literature. There were only three SARS-CoV and six MERS-CoV vaccine clinical trials (Table 1), and extensive effort has been made to develop COVID-19 vaccines in response to the current pandemic. Seven representative vaccine clinical trials were presented in Table 1, including inactivated whole virus vaccine and S protein-derived vaccine. Well-established vaccines targeting pathogens other than SARS-CoV-2 are also under investigation, such as measles (NCT04357028) and BCG (NCT04327206), which may induce strong immune responses and provide non-specific protective effects against SARS-CoV-2 infection (21).

There are two primary design strategies for coronavirus vaccine development: the usage of the whole virus or genetically engineered vaccine antigens that can be delivered through different formats. The whole virus vaccines include inactivated (22) or live-attenuated vaccines (23, 24) (Table 2). The two live attenuated SARS vaccines mutated the exoribonuclease and envelop protein to reduce the virulence and/or replication capability of the SARS-CoV. Recent works also showed promising development of three types of SARS-CoV-2 vaccines, including inactivated whole virus vaccine (25), RNA vaccine (26), and virus-like particles (VLP) vaccine (27) (Table 2). Overall, the whole virus vaccines can induce a strong immune response and protect against coronavirus infections. Genetically engineered vaccines that target specific coronavirus proteins are often used to improve vaccine safety and efficacy. The coronavirus antigens such as S protein, N protein, and M protein can be delivered as recombinant DNA vaccine and viral vector vaccine (Table 2).

From experimentally identified immune responses induced by coronavirus vaccines, we found evidence of the protective roles of both antibody and cell-mediated immunity (28, 29). The protective role of the neutralizing antibody to coronavirus S protein has been demonstrated by the experimental result that a passive transfer of the serum from mice immunized with MVA/S to naïve mice reduced the replication of challenged SARS-CoV in the respiratory tract (28). Here the MVA/S is the highly attenuated modified vaccinia virus Ankara (MVA) containing the gene encoding full-length SARS-CoV S protein. The antibodies developed in the mice immunized with MVA/S could also bind to the S1 domain of S and neutralize SARS-CoV *in vitro*. Passive transfer of anti-S neutralizing antibody also offered protection against SARS-CoV (30). However, antibody responses in patients previously infected with respiratory viruses, including SARS-CoV and MERS-CoV, tend to be short-lived (31). Instead, T cell responses are often long-lived by targeting conserved proteins and showed to have a significant correlation in protective immunity against influenza virus infection (32). SARS-CoV-specific memory T cells but not antibody-producing B cells could be detected in patients 6 years after SARS-CoV infection (33). A further study showed that respiratory tract memory CD4^+^ T cells specific for an epitope the nucleocapsid (N) protein of SARS-CoV provided protection against virulent challenge with SARS-CoV and MERS-CoV (29). CD8^+^ T cells were also found to be crucial for the clearance of SARS-CoV and MERS-CoV infections (34, 35). Therefore, our vaccine prediction would target those viral antigens with the ability to induce protective neutralizing antibody and/or T cell responses.

### SARS-CoV-2 N Protein Sequence Is Conserved With the N Protein From SARS-CoV and MERS-CoV

We first used the Vaxign analysis framework (16, 20) to compare the full proteomes of seven human coronavirus strains (SARS-CoV-2, SARS-CoV, MERS-CoV, HCoV-229E, HCoV-OC43, HCoV-NL63, and HCoV-HKU1). The proteins of SARS-CoV-2 were used as the seed for the pan-genomic comparative analysis. The Vaxign pan-genomic analysis reported only the N protein in SARS-CoV-2 having high sequence similarity among the more severe form of coronavirus (SARS-CoV and MERS-CoV), while having low sequence similarity among the more typically mild HCoV-229E, HCoV-OC43, HCoV-NL63, and HCoV-HKU1. The sequence conservation suggested the potential of N protein as a candidate for the cross-protective vaccine against SARS and MERS. The N protein was also evaluated and used for vaccine development (Table 2). As a protein inside the viral envelope, the N protein packs the coronavirus RNA to form the helical nucleocapsid in virion assembly. This protein is more conserved than the S protein and was reported to induce a humoral and cellular immune response against coronavirus infections (36). A conserved CD4^+^ T cell epitope in the SARS-CoV N was also found important for the induction of protection against the challenge of SARS-CoV or MERS-CoV (29). However, a study also showed the linkage between N protein and severe pneumonia or other serious liver failures, suggesting N protein-induced pathogenesis and possible adverse effects caused by N protein-derived vaccines (37).

### Six Adhesive Proteins in SARS-CoV-2 Identified as Potential Vaccine Targets

The Vaxign RV analysis predicted six SARS-CoV-2 proteins (S protein, nsp3, 3CL-PRO, and nsp8-10) as adhesive proteins (Table 3). Adhesin plays a critical role in the virus adhering to the host cell and facilitating the virus entry to the host cell (38), which has a significant association with the vaccine-induced protection (39). In SARS-CoV-2, S protein was predicted to be adhesin, matching its primary role in virus entry. The structure of SARS-CoV-2 S protein was determined (40) and reported to contribute to the host cell entry by interacting with the angiotensin-converting enzyme 2 (ACE2) (41). Besides S protein, the other five predicted adhesive proteins were all non-structural proteins. In particular, nsp3 is the largest non-structural protein of SARS-CoV-2 comprises various functional domains (42).

**Table 3:** Vaxign-ML prediction and adhesin probability of all SARS-CoV-2 proteins.

		**Protein**	**Vaxign-ML score**	**Adhesin probability**
orf1ab	nsp1	Host translation inhibitor	79.312	0.297
	nsp2	Non-structural protein 2	89.647	0.319
	nsp3	Non-structural protein 3	95.283^*^	**0.524**^**#**^
	nsp4	Non-structural protein 4	89.647	0.289
	3CL-PRO	Proteinase 3CL-PRO	89.647	**0.653**^**#**^
	nsp6	Non-structural protein 6	89.017	0.320
	nsp7	Non-structural protein 7	89.647	0.269
	nsp8	Non-structural protein 8	90.349^*^	**0.764**^**#**^
	nsp9	Non-structural protein 9	89.647	**0.796**^**#**^
	nsp10	Non-structural protein 10	89.647	**0.769**^**#**^
	RdRp	RNA-directed RNA polymerase	89.647	0.229
	Hel	Helicase	89.647	0.398
	ExoN	Guanine-N7 methyltransferase	89.629	0.183
	NendoU	Uridylate-specific endoribonuclease	89.647	0.254
	2′-O-MT	2′-O-methyltransferase	89.647	0.421
	S	Surface glycoprotein	97.623^*^	**0.635**^**#**^
	ORF3a	ORF3a	66.925	0.383
	E	Envelope protein	23.839	0.234
	M	Membrane glycoprotein	84.102	0.282
	ORF6	ORF6	33.165	0.095
	ORF7	ORF7a	11.199	0.451
	ORF8	ORF8	31.023	0.311
	N	Nucleocapsid phosphoprotein	89.647	0.373
	ORF10	ORF10	6.266	0.0

* *Denotes Vaxign-ML predicted vaccine candidate*.

# *Denotes predicted adhesin. Bold value denotes Vaxign-ML predicted vaccine candidate and/or predicted adhesin*.

### Three Adhesin Proteins Were Predicted to Induce Strong Protective Immunity

The recently published Vaxign-ML pipeline was applied to compute the protegenicity (protective antigenicity) score and predict the induction of protective immunity by a vaccine candidate (20). Vaxign-ML predicts the protegenicity score using an optimized supervised machine learning model with manually annotated training data consisted of bacterial and viral protective antigens. These protective antigens were tested to be protective in at least one animal challenge model (43). The performance of the Vaxign-ML models was evaluated (Table S1 and Figure S1), and the best performing model had a weighted F1-score and Matthew's correlation coefficient of 0.94 and 0.66, respectively, in nested cross-validation. Using the optimized Vaxign-ML model, we predicted three proteins (S protein, nsp3, and nsp8) as vaccine candidates with significant protegenicity scores (Table 3). The S protein was predicted to have the highest protegenicity score, which is consistent with the experimental observations reported in the literature. The nsp3 protein is the second most promising vaccine candidate besides S protein. There was currently no study of nsp3 as a vaccine target. The structure and functions of this protein have various roles in coronavirus infection, including replication and pathogenesis (immune evasion and virus survival) (42). Therefore, we selected nsp3 for further investigation, as described below.

### Nsp3 as a Vaccine Candidate

**Figure 1:**
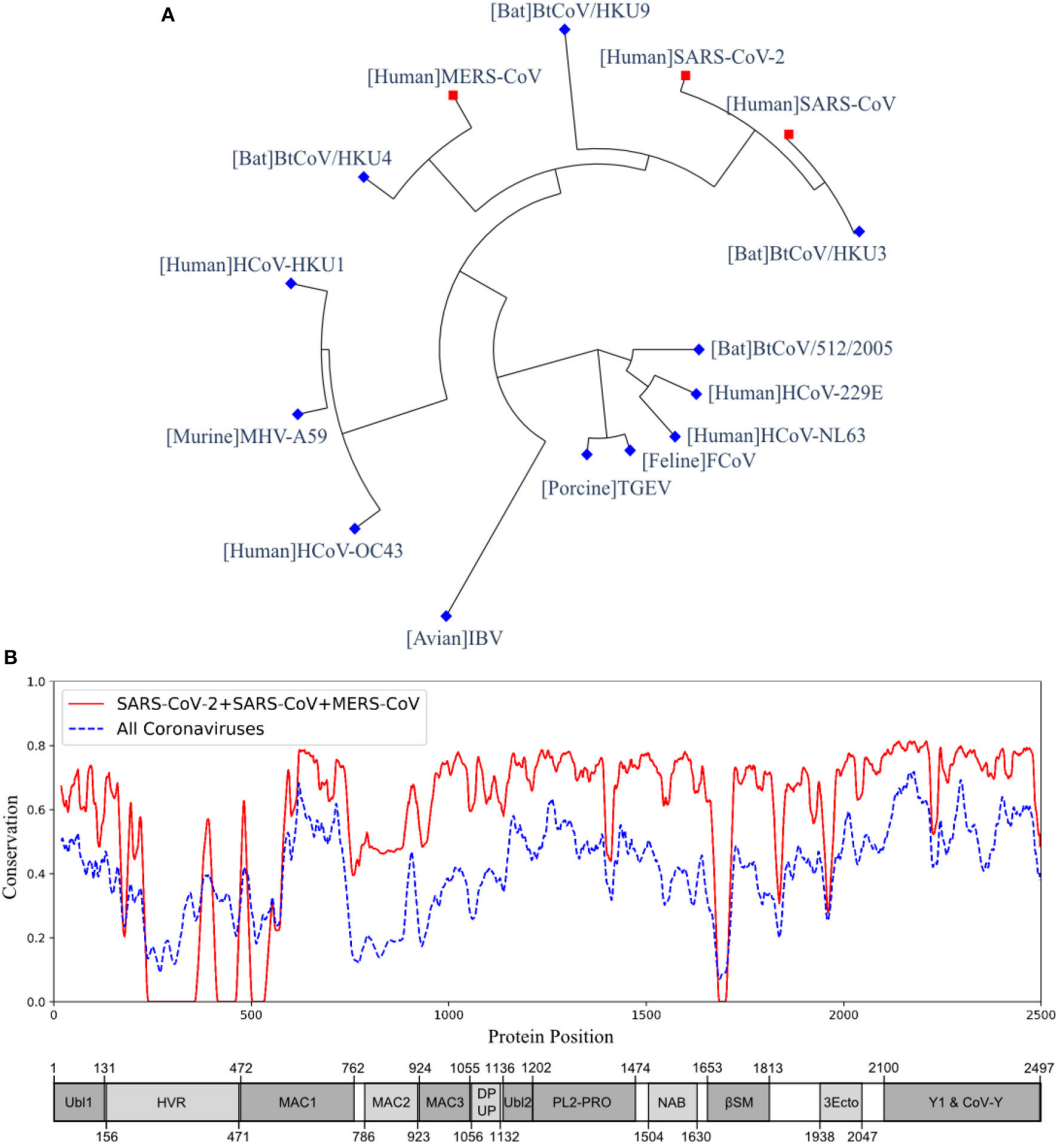
The phylogeny and sequence conservation of coronavirus nsp3. **(A)** Phylogeny of 15 strains based on the nsp3 protein sequence alignment and phylogeny analysis. **(B)** The conservation of nsp3 among different coronavirus strains. The red line represents the conservation among the four strains (SARS-CoV, SARS-CoV-2, MERS, and BtCoV-HKU3). The blue line was generated using all the 15 strains. The bottom part represents the nsp3 peptides and their sizes. The phylogenetically close four strains have more conserved nsp3 sequences than all the strains being considered.

The multiple sequence alignment and the resulting phylogeny of nsp3 protein showed that this protein in SARS-CoV-2 was more closely related to the human coronaviruses SARS-CoV and MERS-CoV, and bat coronaviruses BtCoV/HKU3, BtCoV/HKU4, and BtCoV/HKU9. We studied the genetic conservation of nsp3 protein (Figure 1A) in seven human coronaviruses and eight coronaviruses infecting other animals (Table S2). The five human coronaviruses, SARS-CoV-2, SARS-CoV, MERS-CoV, HCoV-HKU1, and HCoV-OC43, belong to the beta-coronavirus while HCoV-229E and HCoV-NL63 belong to the alpha-coronavirus. The HCoV-HKU1 and HCoV-OC43, as the human coronavirus with mild symptoms clustered together with murine MHV-A59. The more severe form of human coronavirus SARS-CoV-2, SARS-CoV, and MERS-CoV grouped with three bat coronaviruses BtCoV/HKU3, BtCoV/HKU4, and BtCoV/HKU9.

When evaluating the amino acid conservations relative to the functional domains in nsp3, all protein domains, except the hypervariable region (HVR), macro-domain 1 (MAC1) and beta-coronavirus-specific marker βSM, showed higher conservation in SARS-CoV-2, SARS-CoV, and MERS-CoV (Figure 1B). The amino acid conservation between the major human coronavirus (SARS-CoV-2, SARS-CoV, and MERS-CoV) was plotted and compared to all 15 coronaviruses used to generate the phylogenetic of nsp3 protein (Figure 1B). The SARS-CoV domains were also plotted (Figure 1B), with the relative position in the multiple sequence alignment (MSA) of all 15 coronaviruses (Table S3 and Figure S2).

**Figure 2:**
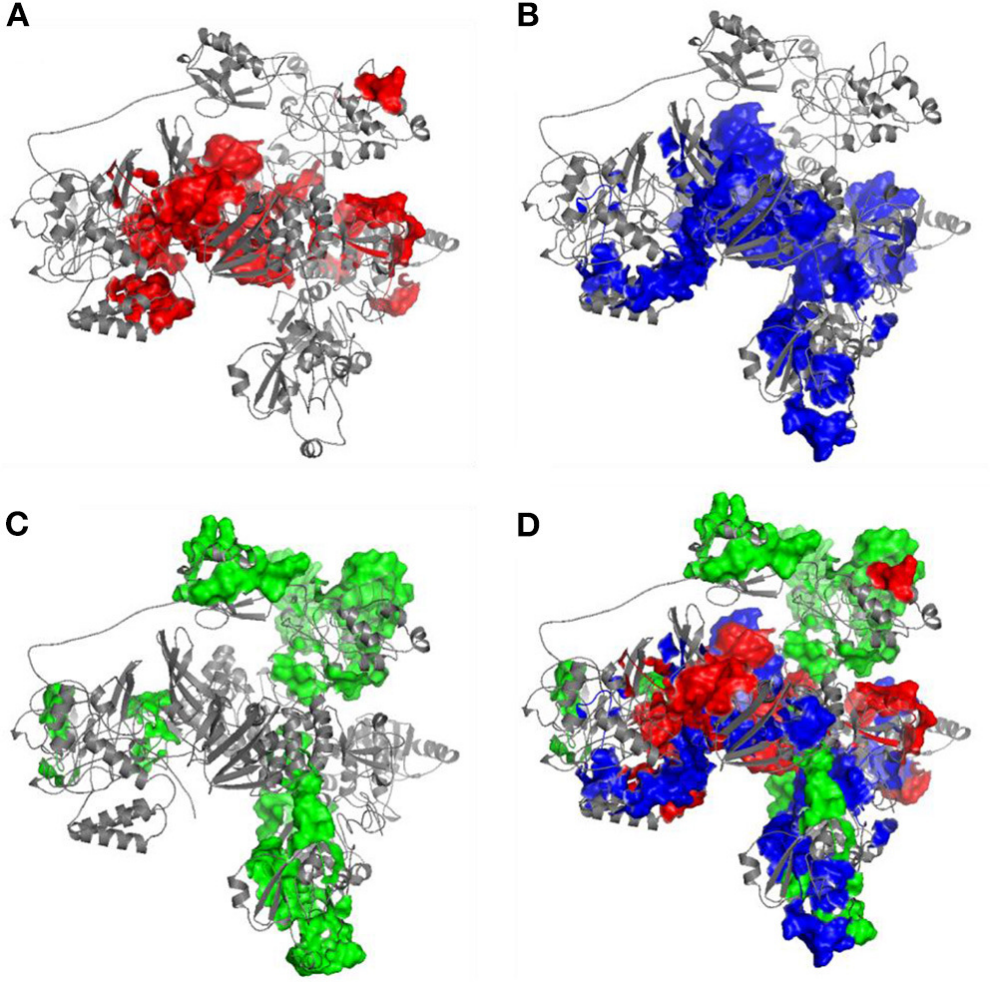
Predicted 3D structure of nsp3 protein highlighted with **(A)** MHC-I T cell epitopes (red), **(B)** MHC-II (blue) T cell epitopes, **(C)** linear B cell epitopes (green), and the **(D)** merged epitopes. The B cell epitopes are more exposed on the protein surface while the T cell MHC-I and MHC-II epitopes are more located within the protein.

**Figure 3:**
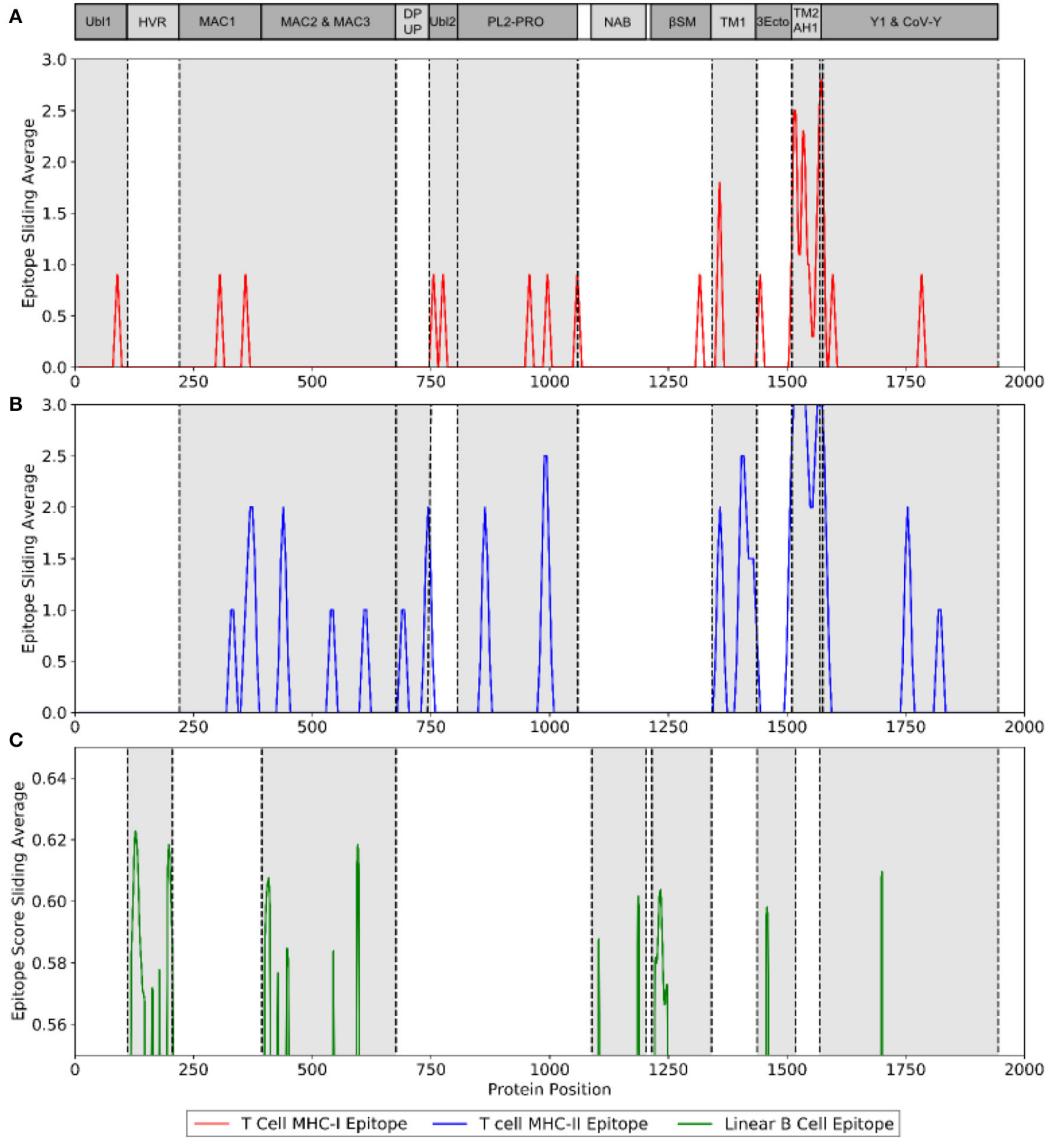
Immunogenic region of nsp3 between SARS-CoV-2 and the four conservation strains. **(A)** MHC-I (red) T cell epitope **(B)** MHC-II (blue) T cell epitope **(C)** linear B cell epitope (green).

The immunogenicity of nsp3 protein in terms of T cell MHC-I & MHC-II and linear B cell epitopes was also investigated. There were 28 and 42 promiscuous epitopes predicted to bind the reference MHC-I & MHC-II alleles, which covered the majority of the world population, respectively (Tables S4, S5). In terms of linear B cell epitopes, there were 14 epitopes with BepiPred scores over 0.55 and had at least ten amino acids in length (Table 4). The 3D structure of SARS-CoV-2 protein was plotted and highlighted with the T cell MHC-I & MHC-II, and linear B cell epitopes (Figure 2). The predicted B cell epitopes were more likely located on the surface of the nsp3 protein. Most of the predicted MHC-I & MHC-II epitopes were embedded inside the protein. The sliding averages of T cell MHC-I & MHC-II and linear B cell epitopes were plotted with respect to the tentative SARS-CoV-2 nsp3 protein domains using SARS-CoV nsp3 protein as a reference (Figure 3). The ubiquitin-like domain 1 and 2 (Ubl1 and Ubl2) only predicted to have MHC-I epitopes. The Domain Preceding Ubl2 and PL2-PRO (DPUP) domain had only predicted MHC-II epitopes. The PL2-PRO contained both predicted MHC-I and MHC-II epitopes, but not B cell epitopes. In particular, the TM1, TM2, and AH1 were predicted helical regions with high T cell MHC-I and MHC-II epitopes (44). The TM1 and TM2 are transmembrane regions passing the endoplasmic reticulum (ER) membrane. The HVR, MAC2, MAC3, nucleic-acid binding domain (NAB), βSM, Nsp3 ectodomain; (3Ecto), Y1, and CoV-Y domain contained predicted B cell epitopes. Finally, the Vaxign RV framework also predicted two regions (position 251-260 and 329-337) in the MAC1 domain of the nsp3 having high sequence similarity to the human mono-ADP-ribosyltransferase PARP14 (NP_060024.2).

**Table 4:** Predicted linear B cell epitopes in nsp3 protein using BepiPred 2.0.

**Epitope**	**Start**	**End**	**Length**
EDEEEGDCEEEEFEPSTQYEYGTEDDYQGKPLEFGATS	111	148	38
EEEQEEDWLDDD	154	165	12
VGQQDGSEDNQ	170	180	11
IVEVQPQLEMELTPVVQTIEV	187	207	21
EVKPFITESKPSVEQRKQDDK	392	412	21
EEVTTTLEETK	419	429	11
YIDINGNLHPDSAT	438	451	14
YILPSIISNEK	536	546	11
RKYKGIKIQEGVVD	586	599	14
DLVPNQPYPNA	1,095	1,105	11
NATNKATYKPNT	1,178	1,189	12
DAQGMDNLACEDLKPVSEEVVENPTIQKDVLECNVK	1,214	1,249	36
YREGYLNSTNVTIA	1,448	1,461	14
GQKTYERHSLS	1,691	1,701	11

## Discussion

Our prediction of the potential SARS-CoV-2 antigens, which could induce protective immunity, provides a timely analysis for the vaccine development against COVID-19. Currently, most coronavirus vaccine studies use the whole inactivated or attenuated virus, or target the structural proteins such as the spike (S) protein, nucleocapsid (N) protein, and membrane (M) protein (Table 2). But the inactivated or attenuated whole virus vaccine might cause strong adverse events. On the other hand, vaccines targeting the structural proteins induce a robust immune response (36, 45, 46). In some studies, these structural proteins, including the S and N proteins, were reported to associate with the pathogenesis of coronavirus (37, 47) and might raise safety concern (12). Recently, the epitopes of the SARS-CoV-2 were computationally predicted and evaluated by sequence homology analysis of SARS-CoV and MERS-CoV epitopes (48). Following this study, the predicted T cell MHC-I and MHC-II epitopes of SARS-CoV-2 was experimentally evaluated using the “megapools” approach and both CD4^+^ and CD8^+^ responses were detected (49). The present work is complementary but not overlapping with the recent reports. Our study applied state-of-the-art Vaxign reserve vaccinology (RV) and Vaxign-ML machine learning strategies to the entire SARS-CoV-2 proteomes, including both structural and non-structural proteins for vaccine candidate prediction. Our results indicate, for the first time, that many non-structural proteins could be used as potential vaccine candidates.

The SARS-CoV-2 S protein was identified by our Vaxign and Vaxign-ML analysis as the most favorable vaccine candidate. First, the Vaxign RV framework predicted the S protein as a likely adhesin, which is consistent with the role of S protein for the invasion of host cells. Second, our Vaxign-ML predicted that the S protein had a high protective antigenicity score. These results confirmed the role of S protein as the important target of COVID-19 vaccines. However, targeting only the S protein may induce high serum-neutralizing antibody titers but cannot induce complete protection (11). In addition, HCoV-NL63 also uses S protein and employs the angiotensin-converting enzyme 2 (ACE2) for cellular entry, despite markedly weak pathogenicity (50). This suggests that the S protein is not the only factor determining the infection level of a human coronavirus. Thus, alternative vaccine antigens may be considered as potential targets for COVID-19 vaccines.

Among the five non-structural proteins being predicted as potential vaccine candidates, the nsp3 protein was predicted to have second-highest protective antigenicity score, adhesin property, promiscuous MHC-I & MHC-II T cell epitopes, and B cell epitopes. The nsp3 is the largest non-structural protein that includes multiple functional domains related to viral pathogenesis (42). The multiple sequence alignment of nsp3 also showed higher sequence conservation in most of the functional domains in SARS-CoV-2, SARS-CoV, and MERS-CoV, than in all 15 coronavirus strains (Figure 1B). Besides the nsp3 protein, our study also predicted four additional non-structural proteins (3CL-pro, nsp8, nsp9, and nsp10) as possible vaccine candidates based on their adhesin probabilities, and the nsp8 protein was also predicted to have a significant protective antigenicity score.

However, these predicted non-structural proteins (nsp3, 3CL-pro, nsp8, nsp9, and nsp10) are not part of the viral structural particle, and all the current SARS/MERS/COVID-19 vaccine studies target the structural (S/M/N) proteins. Although structural proteins are commonly used as viral vaccine candidates, non-structural proteins correlate to vaccine protection. The non-structural protein NS1 was found to induce protective immunity against infections by flaviviruses (51). Since NS1 is not part of the virion, antibodies against NS1 have no neutralizing activity but some exhibit complement-fixing activity (52). However, passive transfer of anti-NS1 antibody or immunization with NS1 conferred protection (53). The anti-NS1 antibody could also reduce viral replication by complement-dependent cytotoxicity of infected cells, block NS1-induced pathogenic effects, and attenuate NS1-induced disease development during the critical phase (54). Finally, NS1 is not a structural protein and the anti-NS1 antibody will not induce antibody-dependent enhancement (ADE), which is a virulence factor and a risk factor causing many adverse events (54). In addition to the induction of antibody responses, non-structural proteins of viruses could induce virus-specific T cells, especially cytotoxic T lymphocytes, that are important to control viral infection. The non-structural proteins of the hepatitis C virus were reported to induce HCV-specific vigorous and broad-spectrum T-cell responses (55). The non-structural HIV-1 gene products were also shown to be valuable targets for prophylactic or therapeutic vaccines (56). Therefore, it is reasonable to hypothesize that the SARS-CoV-2 non-structural proteins (e.g., nsp3) are possible vaccine targets, which might induce cell-mediated or humoral immunity necessary to prevent viral invasion and/or replication.

The SARS-CoV-2 nsp3 protein was recently reported to account for the virus-specific T cell response. Grifoni et al. showed that the three major structural (S/M/N) proteins accounted for 59% of the total CD4^+^ T cell response in COVID-19 recovered patients while other non-structural proteins, including nsp3, also accounted for the response (49). In addition, SARS-CoV-2-reactive CD4^+^ T cells could be detected in a large portion of unexposed individuals, suggesting cross-reactive T cell recognition between SARS-CoV-2 and the other coronaviruses that only cause common cold. In our study, the nsp3 protein showed sequence conservation among the 15 coronaviruses, and particularly, the protein shared higher similarity among the more severe form of coronavirus (SARS-CoV, MERS-CoV, and SARS-CoV-2) (Figure 2). The preexisting immunity against the mild human coronaviruses might offer cross-protection to the SARS-CoV-2 infected individuals (49). In spite of that, none of the non-structural proteins have been evaluated as vaccine candidates, and the feasibility of these proteins as vaccine targets are subject to further experimental verification.

Besides the immunogenicity, safety is also an important factor of a successful COVID-19 vaccine. One of the safety issues of COVID-19 vaccines might occur due to vaccine delivery (e.g., vectors, adjuvants, formulation doses, or route of administration), which cannot be evaluated by the machine learning approach presented in this study. In addition, the nsp3 and other viral adhesive proteins with sequence homology to the host cell adhesion molecules might also cause auto-reactivity with self-antigen or induce T regulatory, leading to low responsiveness of the host to the virus. By applying Vaxign and epitope predictions, our study found that the MAC1 domain of nsp3 protein share sequence homology with the human mono-ADP-ribosyltransferase PARP14, and there is no predicted T cell MHC-I, MHC-II, and linear B cell epitopes within the aligned region.

In addition to vaccines expressing a single or a combination of structural proteins, here we propose an “Sp/Nsp cocktail vaccine” as an effective strategy for COVID-19 vaccine development. A typical cocktail vaccine includes more than one antigen to cover different aspects of protection (57, 58). The licensed Group B meningococcus Bexsero vaccine, which was developed via reverse vaccinology, contains three protein antigens (13). To develop an efficient and safe COVID-19 cocktail vaccine, an “Sp/Nsp cocktail vaccine,” which mixes a structural protein(s) (Sp, such as S protein) and a non-structural protein(s) (Nsp, such as nsp3) could induce more favorable protective immune responses than vaccines expressing a structural protein(s). Current COVID-19 vaccines mostly target on the S protein with various types of delivery systems (such as recombinant virus vectors) (Table 1), and none of the non-structural proteins has not been used. The benefit of a cocktail vaccine strategy could induce immunity that can protect the host against not only the S-ACE2 interaction and viral entry to the host cells, but also protect against the accessary non-structural adhesin proteins (e.g., nsp3), which might also be vital to the viral entry and replication. The usage of more than one antigen allows us to reduce the volume of each antigen and thus to reduce the induction of adverse events. Nonetheless, the potential and safety of the proposed “Sp/Nsp cocktail vaccine” strategy need to be experimentally validated.

For rational COVID-19 vaccine development, it is critical to understand the fundamental host-coronavirus interaction and protective immune mechanism (7). Such understanding may not only provide us guidance in terms of antigen selection but also facilitate our design of vaccine formulations. For example, an important foundation of our prediction in this study is based on our understanding of the critical role of adhesin as a virulence factor as well as protective antigen. The choice of DNA vaccine, recombinant vaccine vector, and another method of vaccine formulation is also deeply rooted in our understanding of pathogen-specific immune response induction. Different experimental conditions may also affect results (59, 60). Therefore, it is crucial to understand the underlying molecular and cellular mechanisms for rational vaccine development.

## Methods

### Annotation of Literature and Database Records

We annotated peer-reviewed journal articles stored in the PubMed database and the ClinicalTrials.gov database. From the peer-reviewed articles, we identified and annotated those coronavirus vaccine candidates that were experimentally studied and found to induce protective neutralizing antibody or provided immunity against virulent pathogen challenge.

### Vaxign and Vaxign-ML Reverse Vaccinology Prediction

The SARS-CoV-2 sequence was obtained from NCBI. All the proteins of six known human coronavirus strains, including SARS-CoV, MERS-CoV, HCoV-229E, HCoV-OC43, HCoV-NL63, and HCoV-HKU1 were extracted from Uniprot proteomes (61). The full proteomes of these seven coronaviruses were then analyzed using the Vaxign reverse vaccinology pipeline (16, 20). The Vaxign program predicted serval biological features, including adhesin probability (62), transmembrane helix (63), orthologous proteins (64), protein functions (16), and Vaxign-ML protegenicity score (20).

The Vaxign-ML protegenicity score was calculated following a similar methodology described in the Vaxign-ML. In brief, the positive samples in the training data included 397 bacterial and 178 viral protective antigens (PAgs) recorded in the Protegen database (43) after removing homologous proteins with over 30% sequence identity. There were 4,979 negative samples extracted from the corresponding pathogens' Uniprot proteomes (61) with sequence dis-similarity to the PAgs, as described in previous studies (65–67). Homologous proteins in the negative samples were also removed. The proteins in the resulting dataset were annotated with biological and physicochemical features. The biological features included adhesin probability (62), transmembrane helix (63), and immunogenicity (68). The physicochemical features included the compositions, transitions, and distributions (69), quasi-sequence-order (70), Moreau-Broto auto-correlation (71, 72), and Geary auto-correlation (73) of various physicochemical properties such as charge, hydrophobicity, polarity, and solvent accessibility (74). Five supervised ML classification algorithms, including logistic regression, support vector machine, k-nearest neighbor, random forest (75), and extreme gradient boosting (XGB) (76) were trained on the annotated proteins dataset. The performance of these models was evaluated using a nested 5-fold cross-validation (N5CV) based on the area under receiver operating characteristic curve, precision, recall, weighted F1-score, and Matthew's correlation coefficient. The best performing XGB model was selected to predict the protegenicity score of all SARS-CoV-2 isolate Wuhan-Hu-1 (GenBank ID: MN908947.3) proteins, downloaded from NCBI. The protegenicity score is the percentile rank score from the Vaxign-ML classification model. A protein with higher protegenicity score is considered as stronger vaccine candidate with higher utility toward protection. In addition, using the protegenicity score of 0.9 as a threshold resulted in the highest prediction performance with weighted F1-score = 0.94 in N5CV.

### Phylogenetic Analysis

The protein nsp3 was selected for further investigation. The nsp3 proteins of 14 coronaviruses besides SARS-CoV-2 were downloaded from the Uniprot (Table S2). Multiple sequence alignment of these nsp3 proteins was performed using MUSCLE (77) and visualized via SEAVIEW (78). The phylogenetic tree was constructed using PhyML (79), and the amino acid conservation was estimated by the Jensen-Shannon Divergence (JSD) (80). The JSD score was also used to generate a sequence conservation line using the nsp3 protein sequences from 4 or 13 coronaviruses.

### Immunogenicity Analysis

The immunogenicity of the nsp3 protein was evaluated by the prediction of T cell MHC-I and MHC-II, and linear B cell epitopes. For T cell MHC-I epitopes, the IEDB consensus method was used to predicting promiscuous epitopes binding to 4 out of 27 MHC-I reference alleles with consensus percentile ranking <1.0 score (68). For T cell MHC-II epitopes, the IEDB consensus method was used to predicting promiscuous epitopes binding to more than half of the 27 MHC-II reference alleles with consensus percentile ranking <10.0. The MHC-I and MHC-II reference alleles covered a wide range of human genetic variation representing the majority of the world population (81, 82). The linear B cell epitopes were predicted using the BepiPred 2.0 with a cutoff of 0.55 score (83). Linear B cell epitopes with at least 10 amino acids were mapped to the predicted 3D structure of SARS-CoV-2 nsp3 protein visualized via PyMol (84). The predicted count of T cell MHC-I and MHC-II epitopes, and the predicted score of linear B cell epitopes were computed as the sliding averages with a window size of ten amino acids. The nsp3 protein 3D structure was predicted using C-I-Tasser (85) available in the Zhang Lab webserver (https://zhanglab.ccmb.med.umich.edu/C-I-TASSER/2019-nCov/).

## Data Availability Statement

All datasets generated for this study are included in the article/Supplementary Material.

## Author Contributions

EO and YH contributed to the study design. EO, MW, and AH collected the data. EO performed bioinformatics analysis. EO, MW, and YH wrote the manuscript. All authors performed result interpretation, discussed, and reviewed the manuscript.

## Conflict of Interest

The authors declare that the research was conducted in the absence of any commercial or financial relationships that could be construed as a potential conflict of interest.
